# Increased cost of care and length of stay due to potentially preventable catheter-related infections in US inpatient data, 2022–2023

**DOI:** 10.1017/ash.2026.10801

**Published:** 2026-07-31

**Authors:** Laura Soloway, Tarja Kärpänen, Aaron Guy

**Affiliations:** Solventum, USA

## Abstract

**Objective:**

This study evaluated differences in total cost of care (TCC) and length of stay (LOS) among patients with potentially preventable catheter-related infections (PPCRIs).

**Methods:**

A retrospective cohort analysis was conducted using the Premier Healthcare Database for calendar years 2022 and 2023. PPCRI cases and at-risk non-PPCRI cases were identified using the Solventum Potentially Preventable Complications (PPC) v38 grouper methodology, which identifies complications occurring during inpatient stays while applying clinical exclusions to avoid capturing progression of preexisting conditions. Each PPCRI case was matched to an at-risk non-PPCRI case based on age, race, sex, diagnosis-related group, and severity of illness.

**Results:**

PPCRI cases experienced significantly higher TCC and LOS in both study years. Mean TCC for PPCRI cases was approximately $162,000 in 2022 and $187,000 in 2023, compared with $44,000 and $40,000 for non-PPCRI cases, respectively (*P* < .0001). Average excess LOS was 38 days in 2022 and 44 days in 2023 for PPCRI cases, versus 6 and 5 days for non-PPCRI cases (*P* < .0001). LOS exceeded expected values in 97% of PPCRI cases compared with 63% of non-PPCRI cases in both years. Mortality was significantly higher among PPCRI cases (17% vs 6% in 2022; 12% vs 2% in 2023; *P* < .0001). Discharge to home occurred more frequently in non-PPCRI cases (48% vs 21% in 2022; 52% vs 23% in 2023; *P* < .0001).

**Conclusions:**

PPCRIs are associated with substantial excess LOS, which drives markedly higher TCC. Targeted prevention of PPCRIs could yield savings of approximately $100,000 per case.

## Introduction

Hospital-acquired infections (HAIs) remain a substantial burden to patients and healthcare systems, contributing to increased morbidity, mortality, hospital length of stay (LOS), and healthcare costs.^1,2^ Among HAIs, central line-associated bloodstream infections (CLABSIs), a type of intravascular catheter-related or associated infections, are a serious concern due to their substantial impact on patient outcomes and healthcare resources, despite being largely preventable with strict adherence to clinical practice guidelines.^3–7^

Multiple studies have demonstrated the substantial clinical and economic burden associated with CLABSIs. In a meta-analysis of case control and cohort studies, most of which were conducted in intensive care unit settings, CLABSIs were associated with a significantly increased risk of death (odds ratio [OR], 2.75; 95% confidence interval^7^: 1.86 to 4.07) compared to no CLABSIs, and this association remained significant among the studies that were matched using illness severity index (OR, 1.51; 95% CI: 1.08 to 2.09).^3^ Similarly, another meta-analysis reported that, compared with uninfected patients, CLABSIs were associated with higher odds of mortality (OR, 3.19; 95% CI: 2.55 to 4.16) and a longer hospital LOS, with a mean difference of 16.14 days (95% CI: 9.27 to 23.01).^4^ In a retrospective study of acute-care hospital patients, a case-matched analysis comparing patients with CLABSIs to a control cohort found that CLABSIs were associated with significantly longer hospital LOS (difference, 15.6–17.4 d), higher costs (an additional $32,759–$55,001), and >3.5-fold increased risk of mortality.^8^ Although surveillance data from the CDC’s National Healthcare Safety Network (NHSN) reported an approximately 13% decrease in CLABSI incidence from 2022 to 2023, this improvement was not observed in long-term acute care hospitals or inpatient rehabilitation facilities, highlighting the continued need for focused infection prevention efforts, especially outside acute care settings.^9^ Evaluating patient outcomes and healthcare resource utilization is therefore essential to inform facility-level decision making in allocating appropriate and often limited resources to improve patient safety, reduce LOS and treatment costs, and ease burden across patients, staff, and healthcare systems.

In addition to their direct clinical and economic impact, CLABSIs contribute to the rising global burden of antimicrobial resistance and the increasing prevalence of untreatable or difficult-to-treat infections, which the World Health Organization has identified as requiring urgent action to reduce potentially avoidable antimicrobial treatments through effective infection prevention methods.^10^ As a result, CLABSI surveillance and the reduction of potentially avoidable infections through effective prevention strategies are critical components of antimicrobial stewardship, patient safety initiatives, and quality of care. Although CLABSI surveillance criteria focus specifically on central venous catheters (CVCs), thereby excluding other vascular access devices (VADs) that also carry infection risk, CLABSI remains a widely adopted and internationally validated surveillance metric.^11^ Broader surveillance measures, such as catheter-associated bloodstream infection (CABSI), have been proposed to encompass all types of VADs, and more recently, hospital-onset bacteremia and fungemia (HOB) has been introduced to capture all types of HAIs beyond catheter-related infections.^12^ However, CABSI and HOB surveillance criteria are less established, whereas CLABSI remains the most prevalent and validated surveillance tool.^13^

VAD-related infections are considered largely preventable, given that adherence to evidence-based insertion and maintenance bundles, including full barrier precautions, chlorhexidine skin antisepsis, appropriate hand hygiene, and prompt catheter removal, has been shown to dramatically reduce infection rates.^14^ However, CLABSI or HOB can still occur despite adherence to best practices due to patient-related factors, such as severe underlying illness with immunosuppression, where infections may be attributable to patient’s vulnerability rather than lapses in care. Umscheid et al. (2011) estimated that approximately 65% of CLABSIs in intensive care settings were reasonably preventable with full implementation of evidence-based measures, implying that a portion might not be easily preventable.^15^ Similarly, a recent pilot study of HOB found that while about one-third of bloodstream infections were judged potentially preventable, others were attributed to intrinsic patient factors (eg, neutropenia) and were less avoidable.^16^ Regardless of residual infection risk in high-risk patients, meticulous adherence to central line insertion and maintenance guidelines is essential for all patients, and any CLABSI should trigger prompt examination of potential gaps in care to uphold a goal of zero harm.

Implementing and sustaining effective infection prevention programs, including adequate staffing, training, auditing, and supportive technologies, requires investment of already limited healthcare resources.^17^ Surveilling and quantifying the impact of CLABSIs on patient outcomes and resource utilization (eg, LOS, total cost of care [TCC], and discharge disposition) can support evidence-based decisions on resource allocation to improve patient safety and reduce the burden on limited resources. While prior studies have estimated the clinical and economic burden of CLABSI, many focus on intensive care settings or rely on older data, highlighting the need for contemporary, hospitalwide analyses.^3–5,8,18,19^

Therefore, this study used recent data from the Premier Healthcare Database (PHD) to analyze differences in TCC and LOS among patients with preventable catheter-related infections (PPCRIs) arising as a complication of the acute inpatient stay, used as a proxy for potentially preventable CLABSIs. Because PPCRI requires the qualifying infection not be present on admission (POA), this approach excludes infections tied to outpatient or community management of VADs (eg, dialysis catheters, chemotherapy infusion lines, home parenteral nutrition) that were already present at admission.

## Methods

### Study design and data source

This retrospective, propensity-score matched cohort analysis was performed using the PHD for calendar years 2022–2023. The PHD is a deidentified, claims-based database that represents a sample of all inpatient admissions across all ages and all payors in the United States (U.S.).^20^

The study was considered exempt research under 45 CFR § 46.104(d)(4), as it involved secondary use of de-identified data in compliance with the Health Insurance Portability and Accountability Act (HIPAA) 45 CFR § 164.514. In accordance with this exemption and the principles of the Declaration of Helsinki, informed consent was waived.

### Inclusion/exclusion criteria

The study population included all patients (all ages and all payors) who had an inpatient stay at a facility included in the PHD data set between January 1, 2022 and December 31, 2023. The population was segmented into those who had a PPCRI and those who did not (non-PPCRI). PPCRIs were used as a proxy for potentially preventable CLABSIs and were determined by a clinical grouping software (Solventum CGS Potentially Preventable Complications v.38) that determines eligibility, at risk, and actual cases of PPCRI.^21^ Specifically, patients with central venous catheter-related infections were identified using the PPC grouping software. PPCRIs were assigned to hospital admissions with a LOS greater than one day in which one or more qualifying secondary diagnosis codes for infection due to a central venous catheter were recorded and were not POA, including bloodstream infection, other specified infection, or unspecified infection due to a central venous catheter (ICD-10-CM T80.211A, T80.218A, T80.219A). PPCRI was not assigned to admissions with a LOS less than two days or to admissions in which procedures from defined PPC exclusion groups were present. These exclusion groups included extracorporeal membrane oxygenation and ventricular assist device-related procedures. A complete list of excluded procedure codes is provided in Supplemental Table. This methodology identified complications occurring during an inpatient stay and applies clinical exclusions to avoid assigning complications that reflect progression of a preexisting condition. By restricting identification to infections that were not POA, only CRI/CLABSIs developing during the inpatient stay were captured. Infections already present at admission, including those from outpatient or community vascular access management, were excluded from the PPCRI definition.

### Variables and outcomes

Patient demographics and hospital characteristics were obtained from the PHD. Because the PHD is claims-based, a unique patient may have more than one inpatient admission during the study period. The data set was matched to a Norms file containing expected number of PPCRI complications and LOS for the corresponding All patient refined diagnosis-related group (APR-DRG) and severity of illness (SOI) of the actual case (created from approximately 13 million inpatient sample claims from 13 states reporting POA indicators and surgical procedure dates within State Inpatient Databases [SID] to the Agency for Healthcare Research and Quality [AHRQ]). The period of claims submission is calendar year 2020 and 2021. The same data source was used in calculating the PPC weights which are integrated for scale with the APR DRG v42 HSRV weights.). Outcome measures included average total cost, average LOS, average expected geometric mean LOS, number of cases over expected, average excess days, total excess days, discharge status, and mortality.

#### Statistical analyses

All identified PPCRI cases were matched one to one with non-PPCRI at risk cases using propensity score matching. Matching variables included age, sex, race, diagnosis-related group (DRG), and SOI to isolate the impact of infection on hospital LOS, TCC, and discharge status. Means, frequencies, and standard deviations (SD) were calculated for patient demographics and hospital characteristics as well as outcome measures. Differences between groups were assessed using analysis of variance, χ^2^, and generalized linear models. All analyses were performed using SAS™ 9.4 (SAS Institute Inc., Cary, NC).

## Results

Demographic characteristics for the two matched cohorts (PPCRI vs non-PPCRI) for 2022 and 2023 are detailed in Table 1 and Table 2, respectively. A total of 10,263,750 at-risk cases were identified through the grouper process, including 5,321,782 in 2022 and 4,941,968 in 2023. Among those at-risk cases, 865 cases in 2022 and 575 cases in 2023 were identified as PPCRI cases and matched to cases without PPCRI (but considered at risk by the grouper process) based on age, sex, race, and DRG and SOI at admission, thereby creating a control cohort with similar risk profiles. After matching, the PPCRI and non-PPCRI cohorts in 2022 and 2023 did not differ significantly with respect to patient demographics (age, sex, race, or ethnicity) or hospital location (rural vs urban). However, hospital teaching status remained significantly different, with a higher proportion of PPCRI cases occurring in large teaching hospitals.

**Table 1.** Demographic information for 2022 after matching on admission DRG, admission SOI, sex, race, and age Table 1 long description.

	PPCRI		Non PPCRI		
Variables	N or Mean	% or SD	N or Mean	% or SD	*P*-value
**N**	865		865		
**Patient demographics**					
**Sex**					.7729
Female	418	48.32	424	49.02	
Male	447	51.68	441	50.98	
**Race**					.4116
Asian	18	2.08	10	1.16	
Black	207	23.93	209	24.16	
Other	64	7.40	55	6.36	
Unknown	32	3.70	30	3.47	
White	544	62.89	561	64.86	
**Ethnicity**					.3683
Non-Hispanic	700	80.92	708	81.85	
Unknown	55	6.36	64	7.40	
Hispanic	110	12.72	93	10.75	
**Age**	53.67	21.59	53.63	21.70	.9708
**Hospital demographics**					
Urban locale	807	93.29	787	90.98	.0741
Teaching (Yes)	572	66.13	490	56.65	<.0001
**Number of beds in facility**					<.0001
000–099	22	2.54	50	5.78	
100–199	92	10.64	103	11.91	
200–299	126	14.57	159	18.38	
300–399	119	13.76	113	13.06	
400–499	100	11.56	100	11.56	
500+	406	46.94	340	39.31	

DRG, diagnosis-related group; N, number; PPCRI, Potentially Preventable Central Catheter-Related Infections; SD, standard deviation; SOI, severity of illness.

**Table 2.** Demographic information for 2023 after matching on admission DRG, admission SOI, sex, race, and age Table 2 long description.

	PPCRI		Non-PPCRI		
Variables	N or Mean	% or SD	N or Mean	% or SD	*P*-value
**N**	575		575		
**Patient demographics**					
**Sex**					.6791
Female	271	47.13	264	45.91	
Male	304	52.87	311	52.87	
**Race**					1
Asian	17	2.96	15	2.61	
Black	164	28.53	166	28.87	
Other	39	6.78	41	7.13	
Unknown	18	3.13	16	2.78	
White	337	58.61	337	58.61	
**Ethnicity**					.4514
Non-Hispanic	469	81.57	465	80.87	
Unknown	46	8.0	37	6.43	
Hispanic	60	10.43	73	12.70	
**Age**	52.27	22.50	52.45	22.03	.8884
**Hospital demographics**					
Urban locale	537	93.39	534	92.87	.7266
Teaching (Yes)	385	66.96	338	58.78	.0041
**Number of beds in facility**					<.0001
000–099	14	2.43	18	3.13	
100–199	16	10.61	73	12.70	
200–299	65	11.30	109	18.96	
300–399	68	11.83	75	13.04	
400–499	76	13.22	72	12.52	
500+	291	50.61	228	39.65	

DRG, diagnosis-related group; N, number; PPCRI, Potentially Preventable Central Catheter-Related Infections; SD, standard deviation; SOI, severity of illness.

All outcome measures were statistically significant after matching, except for average expected LOS, and are detailed for 2022 and 2023 in Table 3 and Table 4, respectively. In 2022, the average patient TCC was $44,126.68 (standard deviation^16^ = $79,918.84) for the non-PPCRI cohort and $162,400.72 (SD = $230,736.01) for the PPCRI cohort (*P* < .0001). Similarly, in 2023, the average patient TCC was $40,416.44 (SD = $62,428.92) for the non-PPCRI cohort and $187,065.72 (SD = $278,274.67) for the PPCRI cohort (*P* < .0001).

**Table 3.** Outcome variables for 2022 after matching on admission DRG, admission SOI, sex, race, and age Table 3 long description.

	PPCRI		Non-PPCRI		
Variables	N or Mean	% or SD	N or Mean	% or SD	*P*-value
**Average patient total cost**	$162,400.72	$230,736.01	$44,126.68	$79,918.84	<.0001
**Average LOS**	45.36	53.56	13.02	24.82	<.0001
**Average expected LOS**	10.52	5.23	10.52	5.35	.9812
**Rate of LOS (actual-expected/expected)**	3.77	5.65	0.22	2.05	<.0001
**Number of cases less than expected**	21	2.43	364	42.08	<.0001
**Number of cases over expected**	843	97.46	501	57.92	<.0001
**Average excess days (actual-expected)**	37.89	53.49	5.93	23.87	<.0001
**Total excess days**	32,774.85		5,129.45		
**Discharge status**					<.0001
**Discharged home**	181	20.92	417	48.21	
**Discharged to other facility**	510	58.96	380	43.93	
**Inpatient**	1	0.12	1	0.12	
**Died**	149	17.23	50	5.78	<.0001
**Left AMA/No available information**	24	2.77	17	1.97	

AMA, Against Medical Advice; DRG, diagnosis-related group; N, number; PPCRI, Potentially Preventable Central Catheter-Related Infections; SD, standard deviation; SOI, severity of illness.

**Table 4.** Outcome variables for 2023 after matching on admission DRG, admission SOI, sex, race, and age Table 4 long description.

	PPCRI		Non-PPCRI		
Variables	N or Mean	% or SD	N or Mean	% or SD	*P*-value
**Average patient total cost**	$187,065.72	$278,274.67	$40,416.44	$62,428.92	<.0001
**Average LOS**	50.8	59.98	11.82	18.34	<.0001
**Average expected LOS**	9.80	4.58	9.87	4.85	.8007
**Rate of LOS (actual-expected/expected)**	4.6	6.38	0.23	1.44	<.0001
**Number of cases less than expected**	13	2.26	210	36.52	<.0001
**Number of cases over expected**	562	97.74	365	63.48	<.0001
**Average excess days (actual-expected)**	43.98	59.46	5.27	17.41	<.0001
**Total excess days**	25,288.5		3,030.25		
**Discharge status**					<.0001
**Discharged home**	133	23.13	297	51.65	
**Discharged to other facility**	359	62.43	252	43.83	
**Inpatient**	0	0	0	0	
**Died**	73	12.7	13	2.26	<.0001
**Left AMA/No available information**	10	1.74	13	2.26	

AMA, against medical advice; DRG, diagnosis-related group; N, number; PPCRI, Potentially Preventable Central Catheter-Related Infections; SD, standard deviation; SOI, severity of illness.

In 2022, the average LOS was 13.02 (SD = 24.82) days for the non-PPCRI cohort and 45.36 (SD = 53.56) days for the PPCRI cohort (*P* < .0001). In 2023, the average LOS was 11.82 (SD = 18.34) days for the non-PPCRI cohort and 50.8 (SD = 59.98) days for the PPCRI cohort (*P* < .0001). The average expected LOS was 10.52 days for both cohorts in 2022 and 9.87 days for the non-PPCRI cohort versus 9.80 days for the PPCRI cohort in 2023; these differences were not statistically significant. The rate for LOS, calculated as (actual LOS-expected LOS)/expected LOS, was 0.22 (SD = 2.05) for the non-PPCRI cohort and 3.77 (SD = 5.65) for the PPCRI cohort (*P* < .0001) in 2022. In 2023, the rate for LOS was 0.23 (SD = 1.44) for the non-PPCRI cohort and 4.6 (SD = 6.38) for the PPCRI cohort (*P* < .0001). Average excess LOS days, calculated as the actual LOS-expected LOS, were 5.93 (SD = 23.87) days for the non-PPCRI cohort and 37.89 (SD = 53.49) days for the PPCRI cohort in 2022 (*P* < .0001). Similarly, in 2023, average excess LOS days were 5.27 (SD = 17.41) days for the non-PPCRI cohort and 43.98 (SD = 59.46) days for the PPCRI cohort (*P* < .0001).

Examination of discharge status in 2022 showed that a substantially higher proportion of patients in the non-PPCRI cohort were discharged home compared with the PPCRI cohort (48.21% vs 20.92%; *P* < .0001). Additionally, the in-hospital mortality rate was significantly higher in the PPCRI cohort than in the non-PPCRI cohort (17.23% vs 5.78%; *P* < .0001). Similar patterns were observed in 2023 for both discharged status and in-hospital mortality (*P* < .0001).

## Discussion

In this large, hospitalwide analysis of more than 10 million at-risk inpatient cases across two consecutive years, PPCRIs were consistently associated with significantly worse clinical outcomes and greater healthcare resource utilization compared with matched at-risk controls without PPCRIs. Patients with PPCRIs, compared with those without PPCRIs, experienced significantly higher total costs (approximately 3–5 fold higher), longer hospital stays (45–51 d vs 12–13 d), greater excess LOS beyond what would be expected (38–44 d vs 4–6 d), and higher in-hospital mortality (13%–17% vs 2%–6%). Although PPCRIs represented a relatively small proportion of at-risk cases, their disproportionate impact on resource utilization and patient outcomes highlights the importance of targeted, hospitalwide infection prevention initiatives. These results align with and extend the findings from recent studies in U.S. acute care hospitals, which have similarly reported that CLABSIs are associated with substantial increases in LOS and healthcare costs, as well as elevated mortality risk.^4,8,9,22^

Across both study years, patients with PPCRIs incurred an average TCC that was at least $100,000 higher than that of the non-PPCRI cohort. Our findings indicate that excess hospital days associated with a PPCRI, which were as high as 44 days, were the primary driver of the increased TCC, as average costs per hospital day did not differ significantly between PPCRI and non-PPCRI cohorts. Additionally, this study captured costs incurred during the acute inpatient stay only, so the true economic burden of PPCRIs is likely underestimated. Downstream costs related to postacute care, including rehabilitation services, long-term care, and potential readmissions, were not captured but are likely substantial, given that a higher proportions of PPCRI patients were discharged to other care facilities rather than home compared with non-PPCRI patients. These results align with prior literature indicating that catheter-related bloodstream infections are among the costliest hospital-acquired complications due to prolonged hospitalization, intensive treatments, and subsequent complications.^1,5,8,15^ The magnitude and consistency of excess costs observed in 2022 and 2023 suggest that the economic burden of PPCRIs remain substantial in contemporary hospital practice, despite ongoing national efforts to reduce healthcare associated infection rates.^23,24^

In addition to increased resource utilization, PPCRIs were associated with significantly worse clinical and discharge outcomes. Across both study years, the inpatient mortality rate was approximately 3 to 6 times higher among patients with PPCRIs compared to those without. These findings are consistent with a recent retrospective cohort study reporting a relative mortality risk of 3.77 for CLABSI compared to control patients without hospital acquired bloodstream infection, with the adjusted mortality rate of 9% vs 2% for CLABSI and control patient cohorts, respectively.^8^ Furthermore, PPCRI patients were less likely to be discharged home, reflecting greater clinical severity and increased need for postacute care.

Another notable finding was the higher proportion of PPCRI cases occurring in large teaching hospitals. This may reflect issues with staffing, greater patient complexity, higher utilization of CVCs, or more intensive surveillance and documentation practices in teaching institutions. Regardless of the underlying drivers for PPCRIs, this observation underscores the importance of improved infection prevention efforts, even in settings such as academic hospitals with advanced clinical infrastructure and highly trained specialists.

Several limitations should be considered when interpreting these results. This study looked at the administrative claims data and therefore may reflect the lower incidence rate of catheter related infections/CLABSI compared to the NHSN surveillance data. However, this study identified potentially preventable cases of CLABSI, through application of PPCRI algorithm which uses a sophisticated clinical grouping software (CGS) that incorporates DRG logic, SOI, and POA indicators to distinguish between preexisting conditions and complications arising during hospitalization. It is widely used in quality improvement, cost containment, and benchmarking initiatives across healthcare systems. The PPCRI algorithm specifically flags bloodstream infections associated with central venous catheters that occur during hospitalization and are not POA, aligning with national definitions of preventability and surveillance standards. The proportion of at-risk admissions flagged as PPCRI was small (865/5,321,782 in 2022; 575/4,941,968 in 2023). For context, NHSN surveillance estimates approximately 18,100 CLABSIs occur annually across U.S. acute care ICUs and wards,^25^ underscoring that PPCRI, applied within a hospital sample rather than the full NHSN-reporting universe, likely captures only a subset of true preventable CRI/CLABSI burden. This exclusion of POA infections tied to outpatient vascular access management likely contributes to the gap between PPCRI counts and broader CRI/CLABSI surveillance figures, which may capture cases regardless of care setting of origin.

Despite these limitations, this study provides robust, contemporary, hospitalwide evidence of the substantial clinical and healthcare resource burden associated with PPCRIs, used here as a proxy for CLABSIs. By using a large hospital database and applying a matched cohort design, these findings extend prior research that has largely focused on intensive care settings or relied on older data. Collectively, the results underscore the importance of continued surveillance, prevention, and targeted quality improvement efforts to reduce CLABSIs and improve patient safety across diverse hospital settings. Future research efforts should include study of the long-term consequences of PPCRI, as differences in discharge status, especially in patients discharged directly to home, were seen.

## Conclusions

Catheter related infections continue to have a great impact on patients and healthcare facilities. Patients who develop a PPCRI incur significantly greater healthcare utilization, with an average hospital stay of nearly 50 days and costs approximately 3–5 times higher than those of similar patients without infection. A higher proportion of patients with PPCRI are discharged to other care facilities rather than home compared with non-PPCRI patients. Moreover, nearly 1 in 6 patients with PPCRI do not survive hospitalization. For clinicians and hospital administrators, these findings highlight the importance of infection prevention, as each PPCRI avoided could potentially save around $100,000 in healthcare costs and four excess hospital bed days per patient.

## Supporting information

10.1017/ash.2026.10801.sm001 Soloway et al. supplementary material Soloway et al. supplementary material

## Supplementary material

The supplementary material for this article can be found at https://doi.org/10.1017/ash.2026.10801.

## Table 1. Long description

A table comparing demographic characteristics of PPCRI and non-PPCRI cohorts for 2022. The table has 16 rows and 7 columns. Column headers are Variables, PPCRI N or Mean, PPCRI % or SD, Non PPCRI N or Mean, Non PPCRI % or SD, and P-value. Row labels include Patient demographics and Hospital demographics. Row 1: N, 865, 865. Row 2: Sex, Female, 418, 48.32, 424, 49.02, .7729. Row 3: Sex, Male, 447, 51.68, 441, 50.98, .7729. Row 4: Race, Asian, 18, 2.08, 10, 1.16, .4116. Row 5: Race, Black, 207, 23.93, 209, 24.16, .4116. Row 6: Race, Other, 64, 7.40, 55, 6.36, .4116. Row 7: Race, Unknown, 32, 3.70, 30, 3.47, .4116. Row 8: Race, White, 544, 62.89, 5614, 64.86, .4116. Row 9: Ethnicity, Non-Hispanic, 700, 80.92, 708, 81.85, .3683. Row 10: Ethnicity, Unknown, 55, 6.36, 64, 7.40, .3683. Row 11: Ethnicity, Hispanic, 110, 12.72, 93, 10.75, .3683. Row 12: Age, 53.67, 21.59, 53.63, 21.70, .9708. Row 13: Hospital demographics, Urban locale, 807, 93.29, 787, 90.98, .0741. Row 14: Hospital demographics, Teaching (Yes), 572, 66.13, 490, 56.65, <.0001. Row 15: Hospital demographics, Number of beds in facility 000-099, 22, 2.54, 50, 5.78, <.0001. Row 16: Hospital demographics, Number of beds in facility 100-199, 92, 10.64, 103, 11.91, <.0001. Row 17: Hospital demographics, Number of beds in facility 200-299, 126, 14.57, 159, 18.38, <.0001. Row 18: Hospital demographics, Number of beds in facility 300-399, 119, 13.76, 113, 13.06, <.0001. Row 19: Hospital demographics, Number of beds in facility 400-499, 100, 11.56, 100, 11.56, <.0001. Row 20: Hospital demographics, Number of beds in facility 500+, 406, 46.94, 340, 39.31, <.0001.

Navigate back to Table 1..

## Table 2. Long description

A table comparing demographic information for PPCRI and Non-PPCRI groups. The table has 17 rows and 6 columns. Column headers are Variables, PPCRI N or Mean, PPCRI % or SD, Non-PPCRI N or Mean, Non-PPCRI % or SD, and P-value. Row labels include N, Patient demographics (Sex, Race, Ethnicity, Age), and Hospital demographics (Urban locale, Teaching, Number of beds in facility). Row 1: N, 575, 0.01, 575. Row 2: Sex Female, 271, 47.13, 264, 45.91, 0.6791. Row 3: Sex Male, 304, 52.87, 311, 52.87. Row 4: Race Asian, 17, 2.96, 15, 2.61, 1. Row 5: Race Black, 164, 28.53, 166, 28.87. Row 6: Race Other, 39, 6.78, 41, 7.13. Row 7: Race Unknown, 18, 3.13, 16, 2.78. Row 8: Race White, 337, 58.61, 337, 58.61. Row 9: Ethnicity Non-Hispanic, 469, 81.57, 465, 80.87, 0.4514. Row 10: Ethnicity Unknown, 46, 8.0, 37, 6.43. Row 11: Ethnicity Hispanic, 60, 10.43, 73, 12.70. Row 12: Age, 52.27, 22.50, 52.45, 22.03, 0.8884. Row 13: Hospital demographics Urban locale, 537, 93.39, 534, 92.87, 0.7266. Row 14: Hospital demographics Teaching (Yes), 385, 66.96, 338, 58.78, 0.0041. Row 15: Number of beds in facility 000-099, 14, 2.43, 18, 3.13, <.0001. Row 16: Number of beds in facility 100-199, 16, 10.61, 73, 12.70. Row 17: Number of beds in facility 200-299, 65, 11.30, 109, 18.96. Row 18: Number of beds in facility 300-399, 68, 11.83, 75, 13.04. Row 19: Number of beds in facility 400-499, 76, 13.22, 72, 12.52. Row 20: Number of beds in facility 500+, 291, 50.61, 228, 39.65.

Navigate back to Table 2..

## Table 3. Long description

A table comparing patient outcomes between PPCRI and Non-PPCRI groups. The table has 11 rows and 6 columns. The columns are labeled as Variables, PPCRI N or Mean, PPCRI % or SD, Non-PPCRI N or Mean, Non-PPCRI % or SD, and P-value. The rows include variables such as Average patient total cost, Average LOS, Average expected LOS, Rate of LOS (actual-expected/expected), Number of cases less than expected, Number of cases over expected, Average excess days (Actual-Expected), Total excess days, Discharge status, Discharged home, Discharged to other facility, Inpatient, Died, and Left AMA/No available information. Each row provides the mean or number and standard deviation or percentage for both PPCRI and Non-PPCRI groups, along with the P-value indicating statistical significance.

Navigate back to Table 3..

## Table 4. Long description

The table compares outcome measures between PPCRI and Non-PPCRI cohorts for the year 2023. It has 10 rows and 6 columns. The columns are labeled as Variables, PPCRI N or Mean, PPCRI % or SD, Non-PPCRI N or Mean, Non-PPCRI % or SD, and P-value. The rows are labeled with different variables such as Average patient total cost, Average LOS, Average expected LOS, Rate of LOS (actual-expected/expected), Number of cases less than expected, Number of cases over expected, Average excess days (actual-expected), Total excess days, Discharge status, Discharge home, Discharged to other facility, Inpatient, Died, and Left AMA/No available information. Each row provides the mean or number and percentage or standard deviation for both PPCRI and Non-PPCRI cohorts, along with the P-value indicating statistical significance.

Navigate back to Table 4..
