# Supplementary material for: Increased cost of care and length of stay due to potentially preventable catheter-related infections in US inpatient data, 2022–2023
Source: Antimicrob Steward Healthc Epidemiol. 2026 Jul 31;6(1):e232. doi: 10.1017/ash.2026.10801 (PMC13435149; doi:10.1017/ash.2026.10801)
Supplement: Supplementary file 1 [file S2732494X26108018sup001.zip › Supplemental Table 1. PPC-54 Exclusion Procedure Groups and ICD-10-PCS Codes.odt]

# Supplemental Table 1. PPC-54 Exclusion Procedure Groups and ICD-10-PCS Codes
Exclusion Group
Description
ICD-10-PCS Codes
Extracorporeal Membrane Oxygenation (ECMO)
Procedures involving extracorporeal membrane oxygenation, including central and peripheral configurations, with intraoperative and non-intraoperative approaches
5A1522F, 5A1522G, 5A1522H, 5A15A2F, 5A15A2G, 5A15A2H
Ventricular Assist Device and Related Cardiac Support Procedures
Procedures involving implantation, insertion, revision, or replacement of short-term or implantable heart assist systems, including devices involving the heart or thoracic aorta
02HA0QZ, 02HA0RS, 02HA0RZ, 02HA0YZ, 02HA3QZ, 02HA3RS, 02HA3RZ, 02HA3YZ, 02HA4QZ, 02HA4RS, 02HA4RZ, 02HA4YZ, 02HW0YZ, 02HW3RZ, 02HW3YZ, 02HW4YZ, 02RA0LZ, 02RA0MZ, 02WA0QZ, 02WA0RS, 02WA0RZ, 02WA0YZ, 02WA3QZ, 02WA3RS, 02WA3RZ, 02WA3YZ, 02WA4QZ, 02WA4RS, 02WA4RZ, 02WA4YZ, 02WW3RZ, X2HX0F9
Codes listed reflect AHRQ-defined PPC-54 exclusion procedure groups. Admissions with any listed procedure were excluded from PPC-54 assignment.
